# Deciphering a Single‐cell atlas and loss‐of‐function analysis revealing *ABCA7*’s transcriptome insights in Alzheimer’s disease

**DOI:** 10.1002/alz.092265

**Published:** 2025-01-03

**Authors:** Lara De Deyn, Lena Duchateau, Tijs Watzeels, Cristina Vicente, Jasper Van Dongen, Baukje Bijnens, Tim De Pooter, Peter De Rijk, Mojca Strazisar, Wim Vandenberghe, Dietmar Rudolf Thal, Renzo Mancuso, Rosa Rademakers, Fahri Küçükali, Kristel Sleegers

**Affiliations:** ^1^ Complex Genetics of Alzheimer’s Disease group, VIB‐UAntwerp Center for Molecular Neurology, Antwerp Belgium; ^2^ University of Antwerp, Antwerp Belgium; ^3^ Rademakers Lab, VIB‐UAntwerp Center for Molecular Neurology, Antwerp Belgium; ^4^ MIND Lab, VIB‐UAntwerp Center for Molecular Neurology, Antwerp Belgium; ^5^ Neuromics Support Facility, VIB‐UAntwerp Center for Molecular Neurology, Antwerp Belgium; ^6^ Experimental Neurology group, Department of Neuroscience, Leuven Belgium; ^7^ Laboratory for Neuropathology, KU Leuven, Leuven Belgium

## Abstract

**Background:**

*ABCA7*, an important risk gene for AD, encodes a transporter implicated in lipid transport and phagocytosis, and its disruptions have been linked to AD pathogenesis. However, the impact of these mutations on AD risk is complex due to their interaction with a multifaceted transcriptional architecture and cell type‐specificexpression patterns. This study aims to analyze the intricate patterns of *ABCA7* expression across diverse cell types, considering various *ABCA7* genotypes in relation to AD patients and non‐carrier controls, while also exploring the effects of *ABCA7* mutations on transcriptome‐wide gene expression.

**Method:**

To address this complexity, we performed droplet‐based single‐nuclei RNA sequencing on BA10 brain samples (*ABCA7* mutation carriers [n = 9], non‐carrier AD patients [n = 6], and non‐carrier control [n = 8]) using 10x Chromium library preparation followed by sequencing on a NovaSeq6000 platform. Cell type annotation was performed using scSorter. Differential gene expression was analyzed using limma‐voom, and pathway analyses were performed using fgsea in R across cell types and studied groups.

**Result:**

We obtained over 400,000 nuclei across 23 samples. Excitatory neurons had the highest proportion of cells expressing ABCA7. However, the highest average expression of ABCA7 was noted in microglia. We detected numerous differentially expressed genes between carrier and non‐carrier AD patients (pval_FDR_adj < 0.05 & abs(logFC) >1). Pathway analysis revealed a notable decrease in the expression of translation‐related genes in microglia, including those associated with nonsense‐mediated mRNA decay, in individuals carrying the mutation.

**Conclusion:**

In conclusion, our study unveils distinctive patterns of *ABCA7* expression and enriched pathways across major brain cell types, suggesting different roles of ABCA7 in different cell types.

